# UC MSCs Educated Tenon (METn) Stimulates Tendon Regeneration Through Rejuvenation of the Complex and Tendon-Derived Cells (TDCs)

**DOI:** 10.1155/sci/8681205

**Published:** 2025-07-10

**Authors:** Young-joo Yun, Yeasol Kim, Tae Woo Kim, Kee Jeong Bae, Chris Hyunchul Jo

**Affiliations:** ^1^Department of Orthopedic Surgery, SMG-SNU Boramae Medical Center, Seoul National University College of Medicine, Seoul, Republic of Korea; ^2^Department of Translational Medicine, Seoul National University College of Medicine, Seoul, Republic of Korea

**Keywords:** regeneration, rejuvenation, tendon, tendon-derived cells, tenon, umbilical cord derived mesenchymal stem cells

## Abstract

Aging, linked to reduced tendon healing and higher injury susceptibility, is associated to the high incidence of rotator cuff (RC) tears in the elderly. Even after RC repair, disordered neofibrovascular scar tissue often occurs, lowering mechanical strength, and tendon-derived cell (TDC) senescence has been suggested as one of the causes. Age reduces the efficacy of mesenchymal stem cell (MSC) therapy for tendon regeneration. However, certain biomaterial exposure may increase MSC differentiation and paracrine effects. We aimed to develop and evaluate an optimal tenon–MSC complex (TSC) for tendon regeneration and investigate its efficacy and antisenescence mechanisms in aged and degenerated TDCs. We proposed a novel method to isolate a maximum quantity of tenon with col6-rich pericellular matrix (PCM) per gram of tendon, utilizing 2% collagenase. In a fibrin 3D gel culture system, rejuvenated METn (TSC) had higher tenogenic marker expression, collagen fiber quantity, and quality than MSC-only or METc (TDC–MSC complex). METn could repair DNA damage and improve cellular metabolism in senescent TDCs by releasing antisenescence factors. TDCs, which overcomes senescence by the METn_CM treatment, also produced a higher quality tendon matrix. In conclusion, this study demonstrates that rejuvenated and functional TSC significantly enhances tendon regeneration by countering senescence in aged and degenerated TDCs, offering a safe approach to enhance the therapeutic potential of autologous senescent MSCs from the elderly.

## 1. Introduction

The regenerative capacity of numerous tissues is diminished with age and their susceptibility to injury increases [1]. Aged tendons, characterized by structural changes in collagen fibers, decreased mechanical strength, increased glycosaminoglycans (GAGs), and slower healing rates, also exhibit a higher risk of injury [2, 3]. Consequently, the incidence of rotator cuff (RC) tears significantly escalates after the age of 50, with prevalence rates reaching 80% in individuals aged 80 and above. This surge in occurrence is speculated to have a strong association with the aging process [4]. RC tear management can be broadly categorized into two main approaches: nonsurgical treatments and surgical intervention. Nonsurgical treatments include physical therapy, corticosteroid and hyaluronic acid injections, and platelet-rich plasma therapy. Conversely, surgical treatment typically entails debridement of the torn tissue apexes followed by repair [5]. However, following surgical repair of RC, the residual presence of disorganized neofibrovascular scar tissue often compromises mechanical strength. This leads to a high retear rate, ranging from 38% to 90%. It has been observed that age is among the factors contributing to these high retear rates [1, 4, 6]. Tendons are mainly composed of tendon-derived cells (TDCs) like tenocytes and tendon stem/progenitor cells (TSPCs), which are responsible for maintaining tendon homeostasis, biomechanical properties, extracellular matrix (ECM) production, and turnover and they undergo a process of senescence with aging. This leads to a reduction in their proliferative and metabolic activities. Several studies have reported that the senescence of TDCs contributes to impaired tendon healing. For example, aged TDCs exhibit reduced proliferation and metabolic activity [7] and show alterations in ECM gene expression associated with cellular senescence [8]. In addition, increased senescence-associated β-galactosidase activity [9] and the involvement of oxidative stress and inflammatory pathways [10] have been observed in aged tendon tissues. Hence, therapeutic strategies that target TDCs senescence and inactivation to promote tissue regeneration are crucial. Future research is crucial to enhance RC tear treatment and management.

Cell based tissue engineering presents a promising approach to tendon regeneration and various cell types, including tenocytes and mesenchymal stem cells (MSCs), have been proposed as potential agents for tendon repair. Notably, compared to terminally differentiated cells like tenocytes, MSCs exhibit superior proliferative and synthetic activities [11]. MSCs either derived from tendons or from other tissues have received increasing attention over the years for improving tendon healing, with numerous in vitro and in vivo research evidence showing that MSCs can contribute to accelerating and improving the quality of tendon healing [12]. Due to their multipotency, MSCs can directly differentiate into a variety of cells, including osteocytes, chondrocytes, and adipocytes [13]. Moreover, emerging evidence suggests that MSCs exert significant beneficial effects such as antisenescence through the release of growth factors, cytokines, exosomes, and other microvesicles, collectively referred to as “paracrine factors (PFs)” [14–16]. However, the age-dependent reduction in MSC therapeutic potency is a major issue. This is likely related to a concomitant decline in differentiation capacity and variations in PF composition and release with advancing age [17–19]. Due to their enhanced therapeutic potential, human fetal MSCs or MSCs derived from young individuals offer several advantages over adult MSCs [20]. For example, young exosomes of stem cells from human exfoliated deciduous teeth have been shown to possess abundant antiaging signals and can alleviate the senescence phenotype of aged TSPCs and maintain their tenogenic capacity [21]. Thus, over the past decade, regenerative medicine has focused on MSC rejuvenation, which slows aging or reverses to an embryonic state [22–24]. Interestingly, exposure to certain biomaterials, such as cells or ECM, could provide a promising strategy to enhance the age-related diminished differentiation potential and paracrine effects of MSCs, while simultaneously mitigating the effects of aging [25–27]. A recent study found that transplanting MSCs with chondrocytes embedded in pericellular matrix (PCM), which is biochemically and biomechanically distinct from tissue-derived ECM, significantly enhanced cartilage formation ability [28, 29]. Concurrently, in vitro studies show that MSCs cocultured with PCM synthesize greater ECM than MSCs or chondrocytes alone [30]. However, the efficacy of TDCs surrounded by PCM (hereinafter referred to as tenon) as a stimulant to enhance MSC differentiation and paracrine actions for tendon regeneration has not been investigated. Future investigations into this promising approach could yield invaluable insights into more effective tendon regeneration strategies.

Given these factors, our study developed and evaluated an optimum tenon–MSC complex (TSC) for tendon regeneration and examined its efficacy and antisenescence mechanisms in aged and degraded TDCs. We hypothesized that the TSC's antisenescence effects on aged and degraded TDCs could enhance tendon regeneration.

## 2. Materials and Methods

### 2.1. Isolation and Cultivation of Human Umbilical Cord-Derived MSCs (hUC MSCs) and Human TDCs (hTDCs)

hUC MSCs and hTDCs were isolated following the established protocol [31]. Briefly, UCs obtained through cesarean section at delivery were cut into 2–4 mm thick slices. One gram of UC slices were placed on a 150 pi dish and incubated at 37 °C in a 5% CO_2_ incubator for 1 h. Low-glucose Dulbecco's modified eagle's medium (LG-DMEM; HyClone; USA) supplemented with 10% fetal bovine serum (FBS; Cytiva; Korea) and antibiotic solution (100 U/mL penicillin, 100 μg/mL streptomycin, and 0.25 μg/mL amphotericin B; Welgene; Korea) was used as culture medium and the medium was carefully poured into the dish where UC slices were placed. The medium was replaced every 2–3 days and when the cells reached 80% confluency, they were separated, filtered through a 100 μm cell filter (SPL Life Sciences; Korea), and replated at a density of 3333 cells/cm^2^. hTDCs were isolated from supraspinatus tendon (SST) by incubating them in high-glucose DMEM (HG-DMEM; HyClone) containing 0.3% type II collagenase (Worthington Biochemical; USA) and an antibiotic solution for 2 h, with gentle agitation. Subsequently, an equal volume of Dulbecco's phosphate-buffered saline (DPBS; Welgene) was added and the cells were filtered through a 100 μm cell strainer. Afterward, the cells were subjected to two washing steps using HG-DMEM culture medium and were seeded at a density of 2–5 × 10^4^ cells/cm^2^. The medium was replaced every 2–3 days and when the cells reached 60%–80% confluency, they were detached and replated at a ratio of 1:3.

### 2.2. Isolation of Human Tenons

Human SST was washed with DPBS and then subjected to digestion in HG-DMEM containing 0.5%, 2%, or 10% type II collagenase and digested for 45 min at 37°C in a shaking incubator set at 90 rpm. Following digestion, an equal volume of HG-DMEM culture medium was added and centrifugation was performed at 500 × *g*. After centrifugation, the samples were washed twice with DPBS and filtered through a 100 μm cell strainer to isolate the tenon.

### 2.3. Fibrin Gel–Based 3D Culture

Fibrin-based constructs were set up by modifying a reported protocol [32]. Briefly, each well of a six-well plate was coated with ~ approximately 3 mL sylgard (SYLG184; WPI; UK) and fixed to the sylgard layer at 10 mm intervals after polymerization for at least 1 week prior to use. Anchors and plates were sterilized with 70% ethanol for 40 min under UV and then presoaked in 25% FBS–DMEM for 1 day. To manufacture 3D fibrin constructs for MSC differentiation studies, 0.08 × 10^6^ of MSCs, 0.02 × 10^6^ of TDCs, or tenons were suspended into 700 μL of LG-DMEM culture medium, 0.02 U/mL thrombin (REYON PHARMACEUTICAL; Korea), 200 μM aminohexanoic acid (Sigma–Aldrich; 07260; UK), and 10 mg/mL aprotinin (Roche; 10236624001) solution. A total of 300 μL of 5 mg/mL fibrinogen (Sigma–Aldrich; F3879) was then added dropwise and the fibrin gel was left to polymerize at 37°C for 3 h and the constructs were incubated at 37°C in a 5% CO_2_ incubator and harvested at 7 days. A total of the five 3D fibrin construct groups were used for this study: TDC-, Tenon-, MSC-, METc-, and METn-3D. METc is a mixed group of MSCs and TDCs, and METn is a mixed group of MSCs and tenons. To manufacture 3D fibrin constructs for TDC differentiation studies, 0.1 × 10^6^ of TDCs were suspended into 700 μL of HG-DMEM culture medium, 0.2 U/mL thrombin, 200 μM aminohexanoic acid, and 10 mg/mL aprotinin solution. A total of 300 μL of 10 mg/mL fibrinogen was then added dropwise and the fibrin gel was left to polymerize at 37°C for 3 h and the constructs were incubated at 37°C with 5% oxygen and harvested at 28 days. A total of the five 3D fibrin construct groups were used for this study: Control-, Bleomycin-, MSC_CM-, Tenon_CM-, and METn_CM-3D.

### 2.4. Immunofluorescence (IF)

Isolated tenons were fixed with 4% paraformaldehyde in PBS buffer for 20 min, followed by three times washing. After permeabilization by 0.1% triton in PBS for 5 min and washing, tenons were blocked with 1% BSA, 10% goat serum, and 0.3 M glycine in PBS-tween for 1 h and were sequentially incubated with anti-col6 (DSHB; 5C6; diluted 1:50) overnight at 4°C. Tenons were labeled by using mouse Alexa Fluor 488 (Invitrogen; #A21121; diluted 1:200) for 45 min at room temperature. DAPI (Invitrogen; D3571; USA) counterstain was used for visualization of cell nuclei. To confirm METn in a bound state, MSCs were labeled with a fluorescent lipophilic dye according to manufacturer's instructions (Invitrogen; V22885). After mixing dil-labeled MSC and tenon at a ratio of 2:8, IF for col6 was performed as mentioned above.

### 2.5. Preparation and Treatment of the Conditioned Medium (CM)

At the same time as fresh tenon was isolated, fresh UC MSCs were also prepared. A total of the three CM groups were used in this study: MSC_, Tenon_, and METn_CM. The ratio of MSC to tenon was fixed at 8:2, with only eight parts used for MSC only and two parts used for tenon only. The amount of serum-free LG-DMEM used was proportional to the cell number and 1 mL of CM was produced from 0.025 × 10^6^ tenon or 0.1 × 10^6^ MSC after 1 day of culture. The CM was finally obtained after centrifugation at 700 g for 7 min. TDCs treated with 10 μg/mL bleomycin for 3 days and cultured for 2 days with individual CMs were analyzed by cell metabolism, senescence associated-β-galactosidase (SA-β-gal), RT-qPCR, and LA-PCR assays.

### 2.6. Cell Metabolism Assay

1 × 10^4^ TDCs/well were seeded in 96-well plates. Cell metabolism was assessed by WST (Roche Diagnostics; USA). The assay was performed according to the manufacturer's protocol.

### 2.7. SA-β-gal Assay

Cellular senescence was measured using the SA-β-gal staining kit (Cell Signaling Technology; USA) and DAPI fluorescence counterstain according to manufactures instructions. After completion of the staining procedures, total of five fields were randomly taken by fluorescence inverted microscopy (Leica Microsystem; Germany). Under microscopy, the percentage of SA-β-gal positive cells and total cells were counted according to the triplicate experiments.

### 2.8. RNA Isolation, Quantitative Reverse Transcriptase-Polymerase Chain Reaction (RT-PCR), and Real Time qPCR

Total RNA using eCube Tissue RNA mini kit (PhileKorea; Korea) was extracted and reverse transcription and amplification were performed as previously described [33]. Briefly, first-strand cDNA was synthesized in a 50 μL reaction volume from 1 μg total RNA by reverse transcription with SuperScript II (Enzynomics; RT005M; Korea). The qRT-PCR was carried out using a LightCycler480 (Roche; Germany) and TOPreal qPCR 2 × PreMIX (SYBR Green with low ROX; Enzynomics; Korea). It was performed using the following program: 95°C for 15 min, 50 cycles of 95°C for 10 s, and 60°C for 15 s, followed by 72°C for 30 s and a final cooling at 40°C for 30 s. GAPDH was used as an internal control. Relative expression levels of each gene were detected at least three times independently. Data were analyzed by the 2^−*ΔΔ*Ct^ method.

### 2.9. Immunoblotting

Total proteins were separated using 8%–12% SDS/polyacrylamide gels and transferred onto PVDF membranes (Merck Millipore; USA) and blocked with 5% nonfat milk in TBST for 1 h. The membranes were then sequentially incubated with anti-col1, anti-col3, anti-col6, anti-p21, anti-p53 (diluted 1:1000 in 5% nonfat milk, overnight at 4°C) and the secondary antibodies conjugated with horseradish peroxidase (diluted 1:5000 in 5% nonfat milk, 1 h at room temperature). Immune complexes were visualized using D-Plus ECL Pico System (Donginbio; Korea) with LAS-4000 mini-imager (Fujifilm; Japan).

### 2.10. Long Application (LA)-PCR Assay

LA-PCR assay was carried out according to the protocol described by Henderson Daryl [32], Suganya et al. [34] and Bekkers et al. [35]. Genomic DNA extraction was carried out using the PureLink Genomic DNA Mini Kit (Invitrogen; K182001) with the manufacturer's directions. After quantitation by Pico Green (Molecular Probes) in a 96-well plate, gene-specific LA-PCR analysis for measuring DNA damage were performed using TaKaRa Ex Premier DNA Polymerase (TaKaRa; RR370S; USA). LA-PCR was carried out to amplify an 8.6 kb region around the human mitochondrial gene or 13.5 kb region around the human β-globin gene in human genomic DNA using the following primers: 5′-AGCCTCCTTATTCGAGCCGAG-3′ and 5′-TGGGTTTAGTAATGGGGTTTGTGG-3′ for human mitochondria and 5′-CGAGTAAGAGACCATTGTGGCAG-3′ and 5′- GCACTGGCTTAGGAGTTGGACT-3′ for human β-globin. The number of cycles and DNA concentration was standardized in each case before the actual reaction so that the PCR reaction remains in the linear stage of amplification. The final PCR reaction condition was standardized at 94°C-1 min; 98°C-10 s and 68°C-7 min for 30 cycles, 15 ng of DNA template was used in each case. Since amplification of a small region would be independent of DNA damage, a small DNA fragment for each gene (mitochondria and β-globin) was also amplified to normalize amplification of large fragments using the following primers: 5′-AGCCTCCTTATTCGAGCCGAG-3′ and 5′-TAAGGGAGGGTAGACTGTTCAACC-3′ for human mitochondria and 5′-ACACTACTCAGAGTGAGACCCA-3′ and 5′-GTGCTCCCACTCAAGAGATATGG-3′ for human β-globin. The PCR reaction condition was 94°C-1 min; 98°C-10 s, 55°C-15 s, and 68°C-30 s for 30 cycles. The amplified products were then visualized on gels and quantitated with ImageJ.

### 2.11. Histological Analysis

Fibrin-based constructs were fixed with 4% paraformaldehyde and embedded in paraffin. A randomly selected slide was stained with hematoxylin and eosin (H&E) and picrosirius red (PSR). The organization and amount of collagen were evaluated with PSR stained slide under circularly polarized light microscopy at ×200 magnification. The amount of col1 and col3 were also analyzed with immunohistochemistry using anti-col1 and anti-col3 antibodies. Serial sections (4 μm) were cut from each paraffin block, deparaffinized, and rehydrated in a graded alcohol series. After antigen retrieval, the sections were incubated with blocking solution and incubated overnight at 4°C with anti-col1 (1:200) and anti-col3 (1:200) antibodies. After incubation with secondary antibodies (1:500) at room temperature for 1 h, the sections were visualized with DAB kit (DAKO; Denmark).

### 2.12. Statistical Analysis

All data are shown as the means ± standard deviations (SDs) from a representative experiment performed in triplicate. The significances of differences were determined using the independent *t*-test and one-way analysis of variance (ANOVA) with Tukey's test. All statistical analyzes were performed with SPSS software version 20 (SPSS Inc; USA). *p*=0.05 was considered to be statistically significant.

## 3. Results

### 3.1. The Isolated Tenon Using 2% Collagenase Showed a col6-Rich PCM

**Figure 1:**
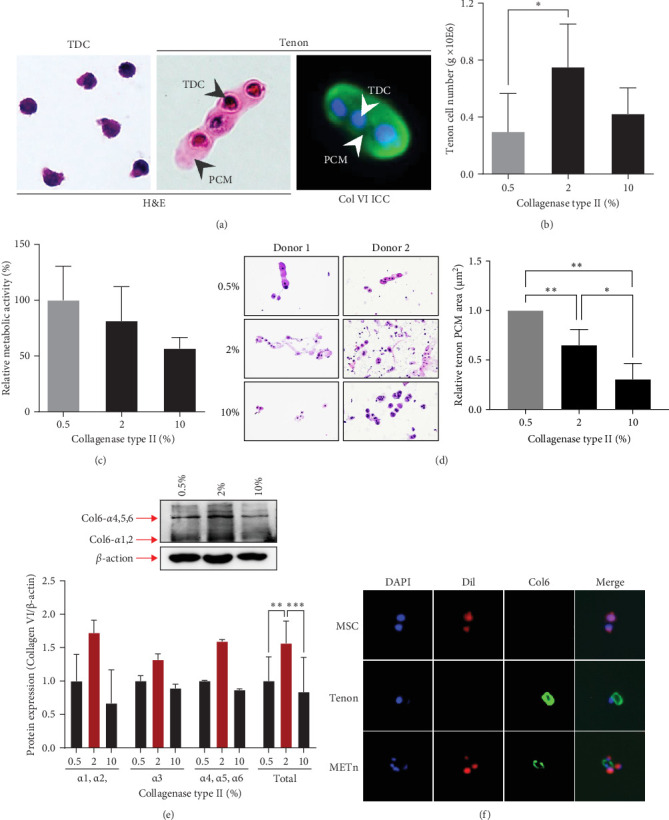
Optimization of collagenase concentration for tenon isolation. (A) Hematoxylin and eosin (H&E) staining and Immunocytochemistry (ICC) analysis. (B) Quantitative assessment of isolated tenon cell numbers were performed to identify variations in cell yield resulting from varying collagenase concentrations: 0.5%, 2%, and 10%. (C) WST assay was carried out to evaluate the effect of collagenase concentration on the metabolic activity and overall health of isolated tenon cells. (D) Measurement of tenon pericellular matrix (PCM) area. (E) Western blotting was employed to analyze collagen type 6 expression within the isolated tenon tissue. (F) DAPI, Dil, and Col6 staining on MSCs, tenon cells, and METn cells, combined with analysis of their direct binding, were performed to investigate potential interactions between these cell types. Data are presented as mean ± SD. Statistical significance: *⁣*^*∗*^*p* < 0.05, *⁣*^*∗∗*^*p* < 0.01, and *⁣*^*∗∗∗*^*p* < 0.001.

According to the results of analysis of PCM components surrounding resident chondrocytes, col6 is the main component of PCM [36]. Therefore, we first confirmed the presence of PCM containing col6 in tenons. ICC and H&E staining showed that col6-rich PCM was present in only tenons, but no such PCM was observed around TDCs (Figure 1A). Then, we compared the number of tenons according to different collagenase concentrations (0.5%, 2%, and 10%). 0.3 million (M), 0.75 M, and 0.42 M tenons were isolated from 1 g of SST at 0.5%, 2%, and 10% concentration, respectively (Figure 1B). Notably, the number of tenon obtained at the 2% concentration was 5.4 times higher than the number of TDCs (0.14 M) isolated from 1 g of SST for 2 h using 0.3% collagenase. Additionally, we assessed the metabolic activity of the tenons using the WST assay, which reflects mitochondrial function as an indirect indicator of cell health. Based on the 0.5% concentration, the metabolic activity of the tenons was 81% at 2% concentration and 57% at 10% concentration (Figure 1C).

We also measured the PCM area and the amount of col6 in tenon at each enzyme concentration. Compared to the 0.5% concentration, PCM area was decreased by 35% (0.65 μm^2^, *p*=0.019) at the 2% concentration and by 69% (0.31 μm^2^, *p*=0.002) at the 10% concentration (Figure 1D). However, the amount of col6 at 2% concentration was 57% higher than 0.5% concentration (*p*=0.02) and 87% higher than 10% concentration (*p*=0.02) (Figure 1E). PCM area narrowed as the enzyme concentration increased, but contrary to expectations, the highest col6 content in tenon was detected was at a 2% enzyme concentration. That is, we confirmed that the highest number of tenons with col6-rich PCM was isolated when using 2% collagenase and these tenons were used in all subsequent experiments.

Prior to all experiments, we confirmed direct binding between the PCM of isolated tenons and dil labeled MSCs (Figure 1F).

### 3.2. MSCs in the Complex State With Tenon Showed Increased Pluripotency

**Figure 2:**
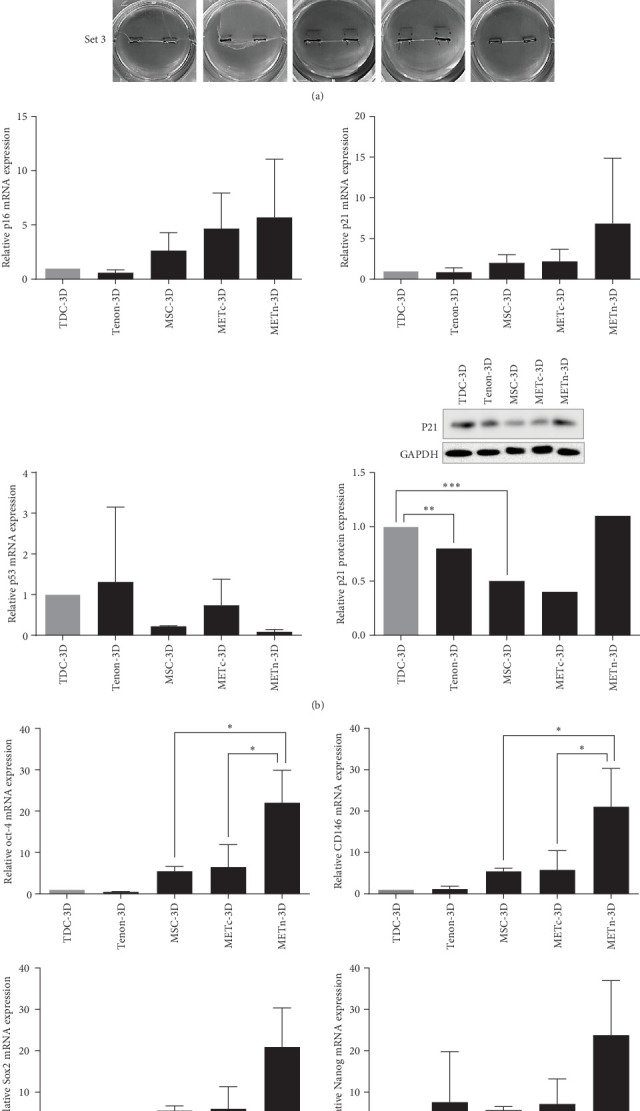
Evaluation of the impact of MSC and tenon cell coculture within 3D fibrin gel. (A) Visual representation of the 3D fibrin gel culture plates utilized in the study, designed to emulate the biomechanical microenvironment of tendons. The experiment included five groups: TDC, tenon, MSC, METc, and METn. (B) Following a 7-day incubation period within the 3D fibrin gel, the mRNA expression levels of key genes associated with cellular senescence, including p16, p21, and p53, were harvested and analyzed. Western blotting was conducted to assess the protein expression of p21. (C) Examination of mRNA expression levels for pluripotency-associated genes, such as Oct-4, Sox2, Nanog, and CD146. Data are presented as mean ± SD. Statistical significance: *⁣*^*∗*^*p* < 0.05, *⁣*^*∗∗*^*p* < 0.01, and *⁣*^*∗∗∗*^*p* < 0.001.

3D fibrin gel culture system was adopted to mimic the microenvironment of the tendon where tensile load, which is important for tendon regeneration, exists. Looking at the morphology after 7 days culture, MSC-3D and METn-3D were similar, whereas METc-3D appeared thinner and more fragile in comparison (Figure 2A).

The mRNA expression of p16 and P21, which are common senescence markers, were increased in METn-3D by 114.7% and 242%, respectively, compared to MSC-3D. At the protein level, p21 was also elevated by 134% in METn-3D compared to MSC-3D. However, mRNA expression of p53, one of important senescence markers in METn-3D was decreased by 56.9% compared to MSC-3D and further by 79% compared to METc, respectively (Figure 2B).

Moreover, mRNA expression of well-known pluripotent markers such as Oct4, Sox2, Nanog, and CD146 in METn-3D were all increased by more than 200% compared to MSC-3D and further by more than 150% compared to METc-3D, respectively (Figure 2C). These data imply that MSCs rejuvenate with reduced senescence and increased pluripotency with the exposure of tenon.

### 3.3. Rejuvenated METn Showed Enhanced Tenogenic Differentiation Potential

**Figure 3:**
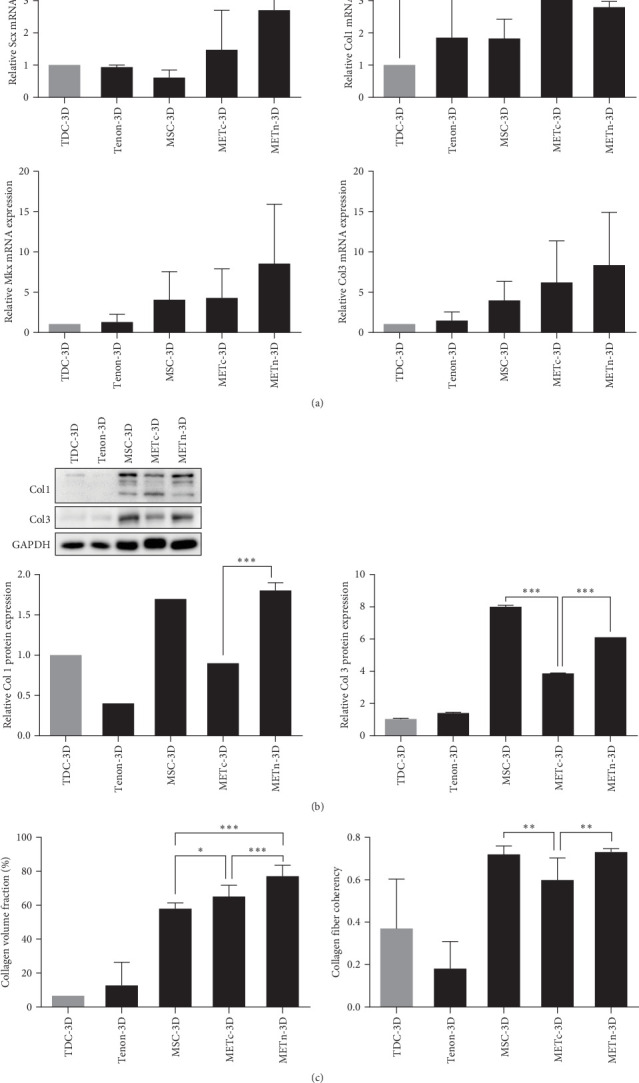
Analysis of gene expression, collagen proteins, and collagen fiber structure following 3D fibrin gel culture. (A) mRNA expression levels of key tendon-related genes, including Scx, Mkx, Col1 and Col3 were examined. (B) Western blotting analysis to evaluate the protein expression of Col1 and Col3, two essential components of the tendon extracellular matrix. (C) Picrosirius red (PSR) histological analysis was employed to investigate collagen fiber structure within the cultured samples. Quantitative measurements of collagen volume fraction and fiber coherency were performed. Data are presented as mean ± SD. Statistical significance: *⁣*^*∗*^*p* < 0.05, *⁣*^*∗∗*^*p* < 0.01, and *⁣*^*∗∗∗*^*p* < 0.001.

Next, we analyzed the tenogenic differentiation potential of METn. It was confirmed that tenogenic transcription factors, Scx and Mkx, in METn-3D, were individually increased by 338.9% and 111.8% compared to MSC-3D, and by 83.6% and 99.6% compared to METc-3D (Figure 3A). In addition to, both col1 and col3 at a protein level, which are important tendon ECM components, were decreased in METc-3D as compared to MSC-3D. However, while col3 was decreased by 23.9% in METn-3D compared to MSC-3D, col1 was increased by 2.9%, suggesting that METn-3D may have improved collagen fiber quality (Figure 3B).

In fact, histologically, it was confirmed that both the quantity and quality of collagen fibers were improved by increasing the collagen volume fraction by 32.9% and the fiber coherency by 56.9% in comparison with MSC-3D (Figure 3C). These data show that rejuvenated METn has the potential to contribute to tendon regeneration by forming quality tendon fibers with improved tenogenic differential potential.

### 3.4. METn Showed Antisenescence Effects on Aged and Degenerated TDCs

Accumulating evidence suggest that injected MSCs exert regenerative effects in vivo, primarily through paracrine effect rather than autonomous differentiation. Therefore, we analyzed their antisenescence effects on bleomycin-induced senescent TDCs using MSC_, Tenon_, and METn_CM. First of all, bleomycin is known to induce not only senescence but also apoptosis, so in this study, we established an in vitro model to induce only senescence without apoptosis according to the purpose of this study. As a result of bleomycin treatment at each concentration for 3 days, typical signs of apoptosis such as floating were not observed up to 50 μg/mL under a microscope. However, while the cell metabolic activity was over 70% up to 20 μg/mL, it began to decrease to below 57% from 25 μg/mL (Figure S1). Further experimental details and supporting data are provided in the supporting information. Therefore, in all senescence related experiments, TDCs were treated with 10 μg/mL bleomycin for 3 days.

**Figure 4:**
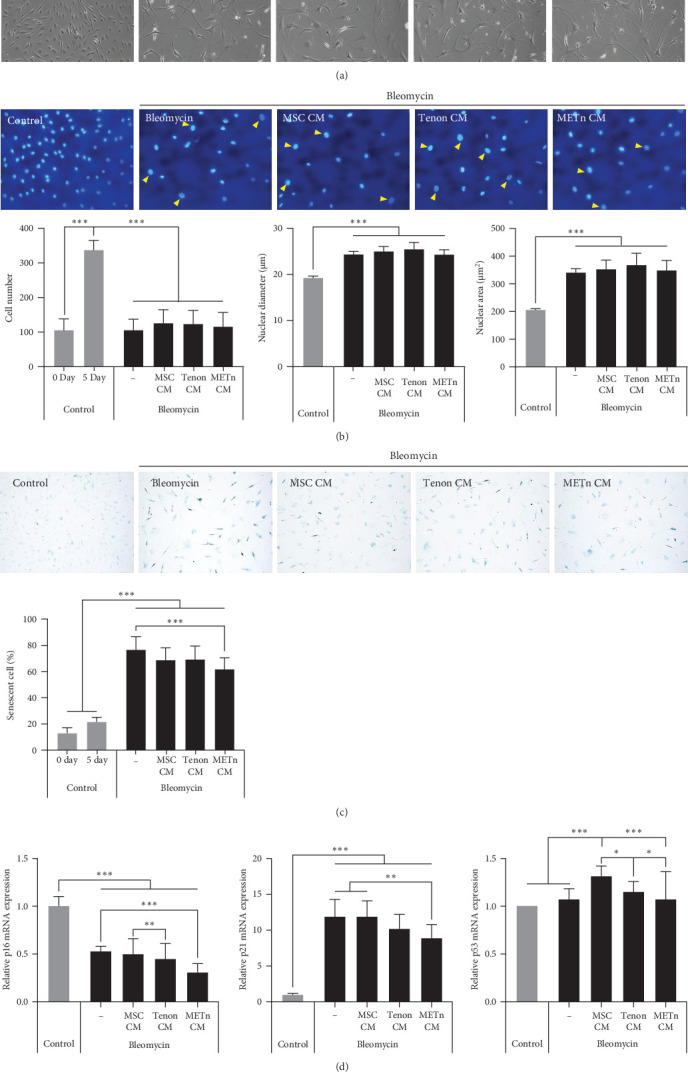
Assessment of antisenescence paracrine effects of MSCs, tenon, and METn_CM on bleomycin-induced senescent TDCs. (A, B) Optical and fluorescence microscopy images depicting morphological changes in senescent TDCs induced by bleomycin treatment. Quantitative analysis included cell number counting, nuclear diameter measurement, and nuclear area assessment. (C) β-galactosidase (β-gal) analysis was performed to quantify the percentage of senescent cells. (D) Evaluation of mRNA expression levels for key senescence-associated genes, including p16, p21, and p53. Data are presented as mean ± SD. Statistical significance: *⁣*^*∗*^*p* < 0.05, *⁣*^*∗∗*^*p* < 0.01, and *⁣*^*∗∗∗*^*p* < 0.001.

Morphological changes such as enlarged and flattened cellular shapes and enlarged nucleus after bleomycin treatment were not restored in all CM groups. Moreover, growth arrest by bleomycin was not significantly recovered in all CM groups (Figure 4A,B).

However, SA-β-gal activity at pH 6.0, which was increased by bleomycin treatment, was decreased by about 10.4%, 9.7%, and 19.5% in MSC_, Tenon_, and METn_CM groups, respectively (Figure 4C). Interestingly, mRNA expression of p16 and P21 were also significantly reduced in METn_CM group by 41.5% and 24.9%, respectively, compared to Bleomycin group. It was confirmed that the protein expression of p21 and P53 was also reduced in METn_CM group compared to Bleomycin group by 9.2% and 50%, respectively (Figure 4D). These data might indicate that METn exerts antisenescence effects on aged and degenerated TDCs.

### 3.5. METn Exhibits an Antisenescence Effect by Repairing DNA Damage and Improving Cellular Metabolism

**Figure 5:**
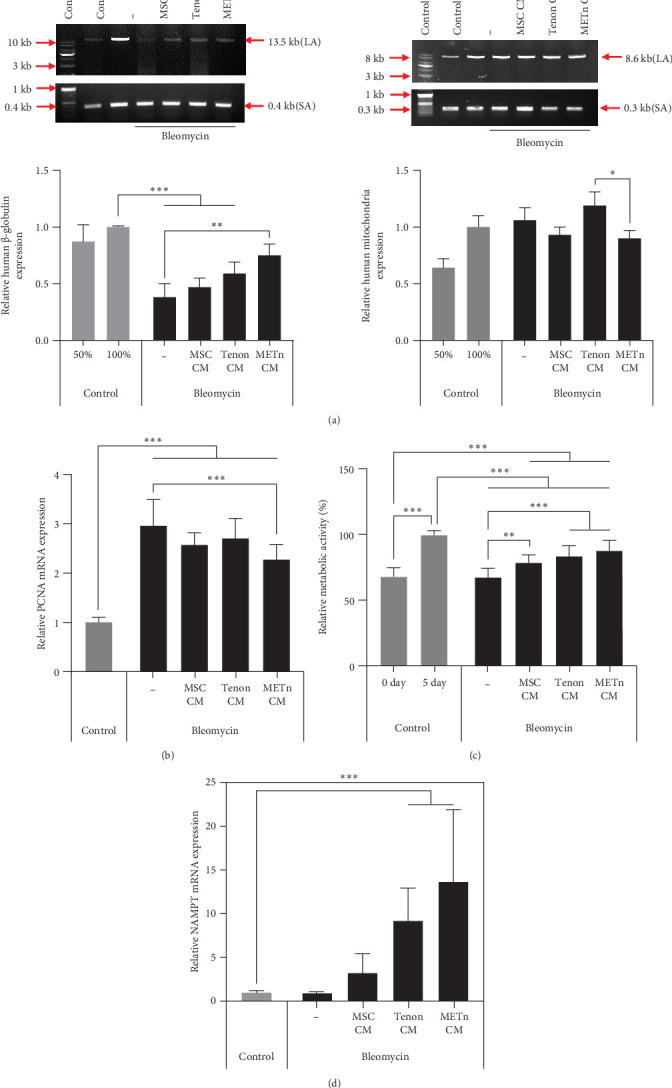
Impact of MSC, tenon, and METn conditioned media on bleomycin-induced DNA damage, PCNA expression, cell metabolism, and NAMPT expression. (A) Long amplicon quantitative PCR analysis was utilized to assess DNA damage, including nuclear and mitochondrial DNA breaks and lesions induced by bleomycin treatment. PCR amplification was performed for both human beta-globulin (nuclear DNA) and human mitochondria (mitochondrial DNA) in the presence of conditioned media (CM) from MSCs, tenon cells, and METn cells. (B) Analysis of proliferating cell nuclear antigen (PCNA) mRNA expression. (C) Cell metabolic activity measurements. (D) Examination of nicotinamide phosphoribosyl transferase (NAMPT) mRNA expression. Data are presented as mean ± SD. Statistical significance: *⁣*^*∗*^*p* < 0.05, *⁣*^*∗∗*^*p* < 0.01, and *⁣*^*∗∗∗*^*p* < 0.001.

Bleomycin is known to cause cell senescence by inducing DNA lesions by single- and double-strand breaks and reducing metabolism by inducing reactive oxygen species (ROS). To identify nuclear DNA and mitochondrial DNA breaks and lesions induced by bleomycin, we employed long amplicon quantitative PCR analysis, one of the most frequently used techniques for assessing DNA damage.  Figure 5A showed that bleomycin did not induce detectable mitochondrial DNA damage, but did damage 60% of nuclear DNA in senescent TDCs. However, 97.4% of nuclear DNA damage was repaired by METn_CM (Figure 5A). It was confirmed that mRNA expression of PCNA, a DNA repair related marker, was reduced by 23% with METn_CM treatment (Figure 5B).

Then, to check the effect of bleomycin on cellular metabolism, we performed WST assay. Compared to the Day 0 no-bleomycin control, the cell number and metabolic activity appear similar, indicating that apoptosis did not occur. However, when compared to the Day 5 no-bleomycin control, the bleomycin-treated group exhibited reduced proliferative capacity, suggesting an overall decrease in cell metabolism. Nonetheless, all types of CMs significantly increased the cellular activity of bleomycin-treated TDCs. In particular, the cellular activity in METn_CM treated group was significantly increased by 20.3% compared to Bleomycin group (Figure 5C). WST is an assay that confirms the activity of living cells by measuring intracellular nicotinamide adenine dinucleotide (NAD+), which is a coenzyme found in all living cell. Therefore, we identified the mRNA expression of NAMPT, a rate-limiting enzyme in the NAD+ salvage pathway that plays an important role in regulating NAD+ levels. Surprisingly, in Tenon_and METn_CM groups, the expression of NAMPT was significantly increased compared to Bleomycin group, and especially the expression was increased by 1355.3% in METn_CM group (Figure 5D). These data suggest that METn could repair DNA damage and improve cellular metabolism in senescent TDCs by releasing the antisenescence factors.

### 3.6. Rejuvenated TDCs Show Restored Tenogenic Differential Potential

**Figure 6:**
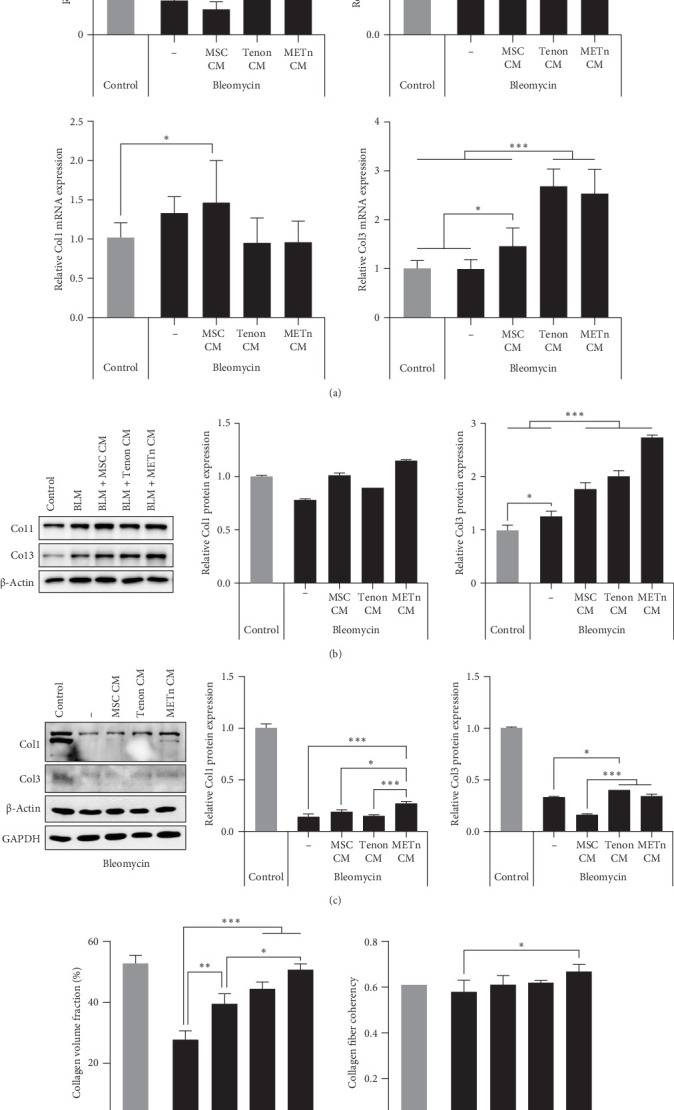
Assessment of tenogenic differentiation potential of rejuvenated TDCs using METn-CM in 2D and 3D culture systems (A) In 2D culture, mRNA expression levels of tenogenic markers, including Scx, Mkx, Col1, and Col3 were examined. (B) Western blotting analysis of Col1 and Col3 protein expression in 2D culture. (C) Western blotting analysis in the 3D culture system further confirmed the protein expression levels of Col1 and Col3. (D) Collagen volume fraction and fiber coherency measurements were conducted. Data are presented as mean ± SD. Statistical significance: *⁣*^*∗*^*p* < 0.05, *⁣*^*∗∗*^*p* < 0.01, and *⁣*^*∗∗∗*^*p* < 0.001.

Finally, we analyzed the tenogenic differential potential of rejuvenated TDCs by METn_CM using 2D-culture or a 3D fibrin gel culture system. In a usual 2D-culture system, it was confirmed that mRNA expression of Scx were increased by 192.9% as compared to Bleomycin group (Figure 6A). Moreover, both col1 and col3 at a protein level were increased in METn_CM group as compared to Bleomycin group (Figure 6B). At the same time, col1 was increased by 92.9% in METn_CM-3D compared to bleomycin-3D, indicating that METn_CM-3D may have improved collagen fiber quality (Figure 6C). In fact, histologically, it was confirmed that both the quantity and quality of collagen fibers were improved by increasing the collagen volume fraction by 83.2% and the fiber coherency by 15.5% in comparison with bleomycin-3D (Figure 6D). These data mean that rejuvenated TDCs by antisenescence paracrine effect of METn could restore tenogenic differential potential.

## 4. Discussion

The most important findings of this study were: (1) 2% collagenase reaction for 45 min isolated a maximum quantity of tenon (0.75 M) with Type VI collagen-rich (75% increase) PCM per gram of tendon compared to 0.5% collagenase reaction; (2) METn was rejuvenated to a younger state with a more than 200% increase of stemness markers Oct4, Sox2, Nanog, and CD146 and a 56.9% decrease of senescence marker p53 at a transcriptional level; (3) rejuvenated METn, tenogenic transcription factors Scx and Mkx were increased by 338.9% and 111.8%, respectively, as well as high-quality collagen fibers with increased collagen volume and fiber coherency were produced; (4) rejuvenated METn's antisenescence factors repaired 97.4% of nuclear DNA damage and improved metabolism by 20.3%, reducing bleomycin-induced premature TDC senescence by 19.5% and improving collagen fiber quality. These findings indicate that rejuvenated TSCs' antisenescence effect on aged and degenerated TDCs could enhance tendon regeneration.

There have been no characterization studies of SST tenons from RC tear surgery have been published. Since Benninghoff introduced chondrons consisting of chondrocytes and PCM in 1925, research has confirmed its biomaterial significance. However, appropriate conditions such as enzyme concentration for isolating chondrons have not been established [28, 30, 37]. Since the maximum time allowed for tenon isolation during METn transplantation from a surgically obtained tendon is 45 min, we fixed the enzyme reaction time at 45 min and compared the number, metabolic activity, PCM area, and Type VI collagen content of tenon separated at three collagenase concentrations. The isolation yield (0.75 M tenons per gram of tendon) was comparable to Bekkers et al. [35] (1.37 M chondrons per gram of cartilage) under the same isolation condition. Moreover, 2% collagenase concentration allows the PCM to contain the most abundant Type VI collagen, which affects the proliferation and differentiation potential of MSCs [38, 39], by eliminating sufficient ECM in the PCM area. At 0.5% concentration, there may be insufficient ECM removal even around the PCM and at 10% concentration, excessive PCM removal may occur, which is likely to result in the relatively highest amount of Type VI collagen at 2% concentration. These results suggest that a 2% collagenase concentration is a suitable condition for isolating the highest number of tenons with Type VI collagen-rich PCM per gram of tendon within the limited time of 45 min.

In MSC therapy for tissue regeneration, MSCs obtained from young donors are more effective, so many methods to transplant young MSCs through stimulation using biomaterials have been suggested [17, 19, 40]. This study compared TDCs and recommended tenon with Type VI collagen-rich PCM as an MSC rejuvenation stimulator. Reprogramming factors Oct4, Sox2, and Nanog as well as CD146 were all more than 200% higher in METn-3D than MSC-3D, but equivalent in METc-3D. In other words, unlike METc, it was clear that METn was in a younger state than MSC monoculture, although it was unclear whether TDCs, MSCs, or both were rejuvenated. These results indicate that Type VI collagen-rich PCM in tenons may stimulate MSCs rejuvenation. There have been studies reveal that intramuscular transplantation of wild-type Type VI collagen^+/+^ fibroblasts improves skeletal muscle stem cell self-renewal in Type VI collagen^−/−^ mutant mice [41]. Type VI collagen promotes proliferation and survival of glioblastoma cells while supporting maintenance of stem-like properties [42]. Type VI collagen has also been shown to enhance MSC proliferation and expansion [38]. This effect is thought to be mediated by its interaction with integrin receptors, which regulate cell adhesion and intracellular signaling pathways associated with self-renewal and differentiation [39, 43, 44]. Given these findings, the presence of Type VI collagen-rich PCM in METn may have contributed to MSC rejuvenation by promoting cell proliferation and enhancing their regenerative potential. It is also possible that the cellular signals of the rejuvenated MSCs are transmitted to TDCs through the PCM [45], thereby rejuvenating TDCs in METn as well. Looking at the reprogramming process of somatic cells, the initial step is “reprogramming-induced senescence or cell cycle arrest,” which is related to DNA damage and metabolic stress caused by ROS generated during the overexpression of pluripotency factors such as Yamanaka. Thus, at this stage, called pre-iPSCs, the expression of tumor suppressor factors such as p21 and p16 genes is activated through activation of p53. In the later stage, the expression and function of these tumor suppressor genes are diminished and endogenous expression of stemness genes such as OCT4, SOX2, NANOG, or other pluripotent genes is induced, leading to complete reprogramming into full-iPSCs. Therefore, p53 could be considered a potent reprogramming barrier that affects the efficacy of reprogramming [46, 47]. In this study, METn-3D lowered P53 expression by 56.9%, a potent reprogramming barrier that influences reprogramming efficacy, while showing an increase in other senescence markers P16 and P21 [48, 49]. Of course, P16 and P21 could be regulated through a P53-independent pathway [50], but it is also possible that METn has just overcome the P53 barrier and has been partially reprogrammed to a younger state. Considering that new cell therapy approaches based on cell reprogramming have a risk of tumor formation, it has recently been proposed that tumor suppressor genes such as p53, p16, and p21 are co-induced with at least one pluripotency factor such as OCT4 or SOX2. This means that METn may have been partially reprogrammed to a younger state rather than transformed into a tumor.

After the growth period of 13–17 years, the core collagen of the tendon is not reproduced [51], so the patient's old and damaged tendon must be rejuvenated to a young tendon composed of TDCs with high healing capacity. In this study, unlike nonrejuvenated METc-3D, which showed decreased expression of tenogenic markers such as col1, col3, and Scx and weak and poor quality of collagen fibers, METn-3D showed significantly improved tendon-like tissue both molecularly and histologically. Rejuvenated METn-3D had 338.9% and 111.8% higher Scx and Mkx levels than MSC-3D. Furthermore, both the quantity and quality of collagen fibers were improved by increasing the collagen volume fraction by 32.9% and the fiber coherency by 56.9%, compared to MSC-3D. Despite much effort, we encountered difficulty in identifying markers that could distinguish TDCs from MSCs [52], so we could not determine which cells in the METn enhances ECM production. According to the report using allogeneic MSCs mixed with autologous chondrons, MSCs stimulate autologous cartilage repair by enhancing the host regenerative response rather than its direct differentiation [29]. From that perspective, MSCs in METn might enhance the ECM synthesis of TDCs in METn rather than their direct differentiation. However, a number of in vitro studies have shown that the differential capacity of MSCs can be improved by coculture with another cell or ECM. For example, tenocytes enhanced the tenogenic differential capacity of MSCs in direct as well as indirect coculture with MSCs [25]. Thus, MSCs or both METn cells may have increased ECM synthesis. However, it was obvious that the ECM production capacity of rejuvenated METn was increased, like neonatal tendons, which show significant increases in tenogenic markers such as Scx and Mkx and stemness markers such as Oct4, Sxo2, and CD146 compared to 6–8-week-old tendons [53]. In other words, this suggests that METn became a young tendon-like tissue with high healing capacity.

MSCs have a significant antisenescence effect by releasing PFs such as exosomes and other microvesicles and younger MSCs are more effective [54–56]. We confirmed that bleomycin-induced senescence of TDSc, which was also attenuated by 10.4% with MSC_CM treatment, was further reduced by 15% with rejuvenated METn_CM treatment with a 50% decrease in senescence marker expression such as P16, P21, and P53. Particularly, bleomycin repaired nuclear DNA damage twofold and increased cell metabolism by 20%, which remained stable. Our findings are comparable to those of human embryonic stem cell-induced MSC (hESC-MSC) derived exosomes, which dose-dependently lower bleomycin-induced DNA damage and HeLa cell viability and intracellular ROS [56]. Therefore, it is assumed that rejuvenated METn also secretes exosomes with components related to these functions. For instance, METn-derived exosomes may contain a great deal of extracellular NAMPT (eNAMPT), which suppresses ROS. A recent study showed that EV-contained eNAMPT enhanced NAD+ biosynthesis after cellular internalization and injection of eNAMPT-containing EVs isolated from young mice not only considerably improved wheel running activity but also extended the lifespan of older mice [57]. Additionally, we found that the ECM production of rejuvenated TDCs by METn_CM was ultimately increased. This suggests that allogenic MSC in rejuvenated METn may not only enhance ECM production of TDCs in the patient's tenon but also improve ECM production of endogenous TDCs after transplantation into the tendon niche of the recipient patient. Therefore, the tissue regeneration-enhancing effect of METn as a safe biomaterial may be more pronounced.

There are some limitations to this study. First, although the effect of METn was confirmed, it is unclear which of the MSCs or tenons in the complex played an important role or whether both together played an important role. Second, this study focused only on the presence and amount of Type VI collagen in PCM, but its role was not elucidated. Furthermore, because a comprehensive analysis of PCM components has not been carried out, it is unclear which components contribute to the functional regulation of METn. According to research results on PCM components in chondrons, PCM is composed mainly of type VI collagen, but also contains high concentrations of proteoglycans, including aggrecan, hyaluronan, and decorin as well as fibronectin and Types II and IX collagen [58, 59]. PCM within tenon is also likely to be composed of these components, which are known to affect proliferation of MSCs and stem cell homeostasis [60, 61]. Third, since comparative analysis of the components of all CMs was not conducted, it is unknown why there was a difference in the antisenescence effect on aged and degenerative TDCs. Fourth, the TDCs used in this study would be a mixture of tenocytes and TSPCs [62]. Because there are no molecular markers that can distinguish between TSPCs and tenocytes, it is difficult to distinguish which cells are responsible for our findings [63]. However, considering that the patient's tendon niche contains both cell types, the results obtained in this study are thought to be meaningful without a clear distinction between two cell types. Fifth, the study lacks experimental data from animal models, making it difficult to directly demonstrate the effectiveness and safety of METn in vivo and its long-term effects on tendon regeneration. Future studies using appropriate animal models will be necessary to validate the therapeutic potential of METn and further investigate its biological mechanisms in a physiologically relevant environment. Last, since the low survival rate after injection is still a problem to be solved in cell therapy, methods that can improve the survival rate of injected METn after RC repair need to be proposed [64].

Taken together, we developed and evaluated an optimal TSC for tendon regeneration and investigated its efficacy and underlying antisenescence mechanisms within the context of aged and degenerated TDCs. We proposed that TSC could significantly enhance tendon regeneration by exerting antisenescence effects on aged and degenerated TDCs.

## 5. Conclusion

In conclusion, our study successfully established an optimal method to isolate tenon with a collagen type 6-rich PCM using 2% collagenase concentration. These tenon cells displayed enhanced metabolic activity and retained a robust PCM structure. Furthermore, we demonstrated that METn, when compared to MSCs alone or METc, exhibited remarkable antisenescence properties, increased pluripotency, tenogenic differentiation potential, and tissue repair mechanisms. METn's rejuvenating effects, ability to counteract senescence, to enhance tenogenic differentiation make it a promising approach for tendon regeneration and age-related tendon issues, advancing regenerative medicine.

## Data Availability

The data that support the findings of this study are available from the corresponding author upon reasonable request.

## Supporting Information

Supporting Information Figure S1. Bleomycin dose titration. (A) Optical microscopy images depicting changes in cellular morphology of cells exposed to varying concentrations of bleomycin. (B) Measurement of cell viability in response to different bleomycin concentrations.
