# Correction to “UC MSCs Educated Tenon (METn) Stimulates Tendon Regeneration Through Rejuvenation of the Complex and Tendon‐Derived Cells (TDCs)”

**DOI:** 10.1155/sci/9842742

**Published:** 2025-12-19

Y. Yun, Y. Kim, T. W. Kim, K. J. Bae, C. H. Jo, “UC MSCs Educated Tenon (METn) Stimulates Tendon Regeneration Through Rejuvenation of the Complex and Tendon‐Derived Cells (TDCs)” *Stem Cells International* 2025 (2025): 8681205 https://doi.org/10.1155/sci/8681205

In the article titled “UC MSCs Educated Tenon (METn) Stimulates Tendon Regeneration Through Rejuvenation of the Complex and Tendon‐Derived Cells (TDCs),” the information in the Funding section was incorrect.

The funding statement should read as follows:

This should have read: “This research was supported by a grant (RS‐2022‐NR070504) of the National Research Foundation of Korea (NRF) funded by the Korea government (MSIT), a grant (22C0608L1) of the Korean Fund for Regenerative Medicine funded by Ministry of Science and ICT, and Ministry of Health and Welfare, a grant (RS‐2023‐00302383) from Technology and Information Promotion Agency funded by Ministry of SMEs and Startups, and a grant (03‐2023‐3080) from the SNUH Research Fund.”

We apologize for this error.

