# Supplementary material for: UC MSCs Educated Tenon (METn) Stimulates Tendon Regeneration Through Rejuvenation of the Complex and Tendon-Derived Cells (TDCs)
Source: Stem Cells Int. 2025 Jul 10;2025:8681205. doi: 10.1155/sci/8681205 (PMC12271689; doi:10.1155/sci/8681205)
Supplement: Supporting Information — Figure S1. Bleomycin dose titration. (A) Optical microscopy images depicting changes in cellular morphology of cells exposed to varying concentrations of bleomycin. (B) Measurement of cell viability in response to different bleomycin concentrations. [file 8681205.f1.pptx]

## Slide 1
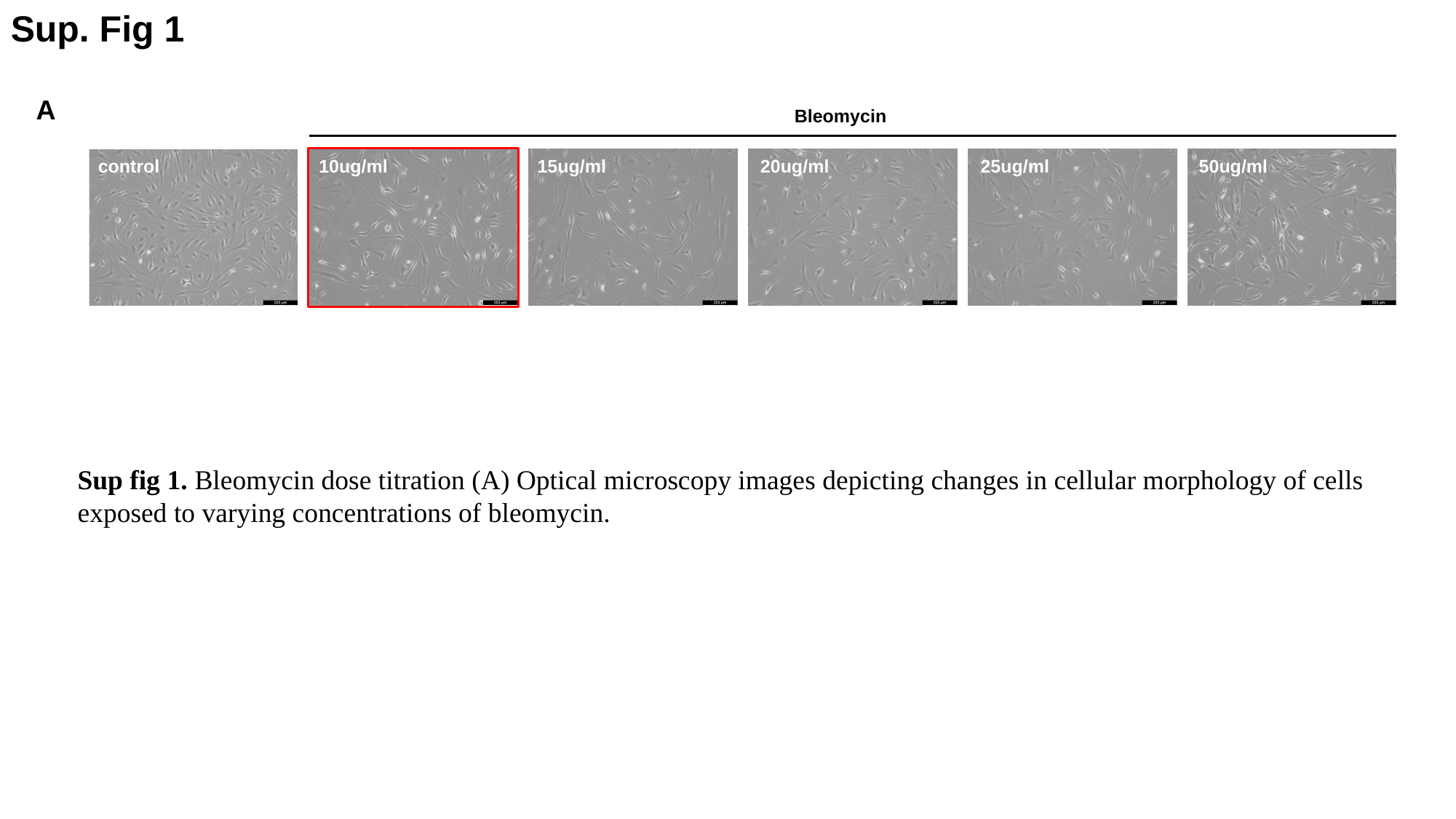

Sup. Fig 1
A
Bleomycin
25ug/ml
50ug/ml
20ug/ml
10ug/ml
15ug/ml
control
Sup fig 1. Bleomycin dose titration (A) Optical microscopy images depicting changes in cellular morphology of cells exposed to varying concentrations of bleomycin.
